# Editor of the Dental Cosmos

**Published:** 1891-07

**Authors:** 


### EDITOR OF THE DENTAL COSMOS.

The death of Dr. James W. White made it necessary to fill the place he for many years so ably occupied. The selection has fallen upon Dr. E. C. Kirk of this city (Philadelphia). Dr. Kirk is well known as a writer in dental periodical literature, and will, without doubt, maintain the high standard which the *Cosmos* has held in the past.

